# Polymorphism in Commercial Sources of Fusidic Acid: A Comparative Study of the* In Vitro* Release Characteristics of Forms I and III from a Marketed Pharmaceutical Cream

**DOI:** 10.1155/2017/3493096

**Published:** 2017-09-17

**Authors:** Jonathan Byrne, Robert Reinhardt, Trinidad Velasco-Torrijos

**Affiliations:** ^1^R&D Department, mibe GmbH Arzneimittel, 06796 Brehna, Germany; ^2^Department of Chemistry, Maynooth University, Maynooth, County Kildare, Ireland

## Abstract

A comparison of the polymorphic forms of 3 commercial sources of fusidic acid using FTIR and XRPD techniques has been performed in this study. It has been demonstrated that polymorphic Forms I and III are currently available on the commercial market. The influence of the observed polymorphism on the stability of the drug substance in bulk form has been investigated through stability and stress testing according to current ICH guidelines. Significant differences were detected between commercial sources with regard to the stability of the bulk substance under photolytic and humidity stress conditions. When properly packaged in an inert atmosphere, fusidic acid from all 3 manufacturers showed a comparable stability. The effects of the observed polymorphic differences on the intrinsic dissolution rate of the drug substance and its* in vitro* release from the marketed drug product Fusicutan® plus Betamethasone cream have been investigated. Results indicated that the release rate of the drug substance is similar for polymorphic Forms I and III, allowing both forms to be used during manufacture without affecting the safety or efficacy of the drug product.

## 1. Introduction

**Figure 1:**
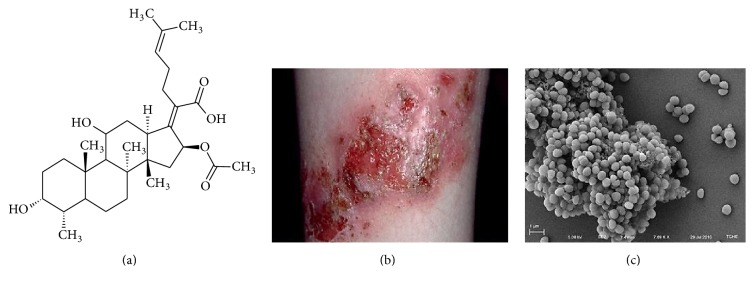
(a) Chemical structure of fusidic acid, (b) an example of infected atopic eczema of the forearm, and (c) a scanning electron micrograph of* Staphylococcus aureus*.

Fusidic acid (FA, Figure 1(a)) is one of 18 naturally occurring triterpene compounds of fungal origin [1] which collectively make up the* Fusidane* group of antibiotic substances [2]. It is the most potent member of this group and is, to date, the only representative to have been used clinically [2]. It was first isolated fermentatively in the early 1960s from the fungus* Fusidium coccineum *[3], with the description of a complete chemical synthesis following some 20 years later [4]. FA is mainly used in the treatment of Gram-positive bacterial infections (e.g., infected atopic dermatitis, Figure 1(b)), particularly those caused by* Staphylococcus *species [2, 5, 6], and functions by binding to prokaryotic elongation factor G (EF-G), effectively stalling the elongation step of bacterial protein synthesis [7–9]. At clinically relevant concentrations, the substance has a bacteriostatic effect but can work as bactericidal at higher concentrations [10]. The past decade has seen renewed interest in this drug as a treatment for methicillin-resistant* Staphylococcus aureus* (MRSA, Figure 1(c)) [11–16] which can in part be attributed to its low toxicity and to the low incidence of resistance to FA in relevant bacterial populations [14, 15].

FA is known to demonstrate polymorphism, with 4 crystalline forms (I–IV) described to date in relevant literature [17–19]. It has been well documented that polymorphs of the same drug substance can possess different solubility properties which may lead to variations in local and/or systemic bioavailability and stability [20–23]. This can present problems when a pharmaceutical manufacturer needs to change supplier of an active pharmaceutical ingredient (API) or add a second supplier in order to ensure a constant supply of a particular drug substance. In such cases, comparative characterisation studies of the crystalline structure, solubility, and* in vitro* release properties of the API from the drug product, as well as associated stability studies, must be performed.

Despite the fact that FA has been on the market for several decades [3], polymorphism in this substance has only been documented in more recent times [17–19] and to date no report has focused on the release rate and stability of different polymorphs of FA from topical pharmaceutical formulations. Strict legislative requirements are in place with regard to the use of different polymorphic drug forms in pharmaceutical products [24]. Thus, the goal of the current study is to assess the effects of polymorphism in commercial sources of FA on (i) the stability of the bulk drug substance and (ii) its* in vitro* release rate from a marketed topical pharmaceutical dosage form. This information is of critical importance when considering whether FA from different commercial sources can be used interchangeably in semisolid pharmaceutical preparations without significant implications for the safety and efficacy of the product.

## 2. Materials and Methods

### 2.1. Reagents

Fusidic acid of Ph. Eur. quality was purchased from Ercros SA (Madrid, Spain, Manufacturer A), Joyang Laboratories (Jiangsu, China, Manufacturer B) and OJSC Biosintez (Penza, Russia, Manufacturer C). Gradient grade methanol and acetonitrile were purchased from VWR International GmbH (Darmstadt, Germany). Purified water was obtained from the in-house purification system at mibe GmbH Arzneimittel (Brehna, Germany). Phosphoric acid (85% m/m, analysis grade, *d* = 1.71 g/ml) was purchased from Merck (Darmstadt, Germany). All cream samples were provided by mibe GmbH Arzneimittel (Brehna, Germany).

### 2.2. Instrumentation and Experimental Procedures

#### 2.2.1. Stability Studies and HPLC Analysis

Stability studies of micronised and nonmicronised FA from each manufacturer were performed in Binder KBF 720 stability chambers (Binder Inc., Tuttlingen, Germany) according to relevant ICH guidelines. The aim of the studies was to investigate if the polymorphic modification of FA influences the stability of the bulk drug substance during storage. It should be noted that the particle size distributions of FA from each manufacturer were comparable. Samples comprising 5 g of drug substance stored under a nitrogen atmosphere in a suitably sealed polyethylene bag, which was further packaged in a heat sealed aluminium sachet, were placed on stability under the conditions of 5 ± 3°C, 25°C/60% relative humidity (RH), 30°C/65% RH, and 40°C/75% RH for 24, 24, 12, and 6 months, respectively.

HPLC experiments were performed with Shimadzu Prominence HPLC Systems (Shimadzu, Japan). The systems were equipped with a binary pump (LC-20AD), a temperature-controlled autosampler (SIL-20AC_HT_), a temperature-controlled column compartment (CTO-20AC), and an online degasser (DGU-20A_5_). A DAD detector (SPD-M20A) was employed. The software packages used were Shimadzu LCsolution version 1.24 SP1 and Shimadzu Class-VP version 6.14 SP2A. Samples were prepared and analysed using the stability-indicating procedure described in the European Pharmacopeia (Ph. Eur.) monograph for fusidic acid hemihydrate [25].

#### 2.2.2. XRPD/ATR-FTIR/SEM

XRPD analysis was performed using a Bruker D8 Advance (Bruker, Massachusetts, USA) with parallel beam geometry and a Cu/Cu K*α* radiation source. Samples were scanned from 5° to 60°2*θ* at a step size of 0.03° and a step time of 1 s using a LynxEye detector. Data was collected and analysed using Bruker Diffrac Plus XRD software. ATR-FTIR analysis was performed on a Perkin-Elmer SpectrumOne spectrophotometer (Perkin-Elmer, Massachusetts, USA). Scanning electron micrographs were obtained using a Hitachi 3200N SEM instrument (Hitachi, Tokyo, Japan).

#### 2.2.3. Intrinsic Dissolution/*In Vitro* Diffusion Test

Intrinsic dissolution experiments were performed using an ERWEKA DT800 dissolution apparatus (ERWEKA, Heusenstamm, Germany) equipped with 6 rotating cylinders as described in Ph. Eur. 2.9.29. A rotation speed of 50 rpm and a medium comprising 500 ml of 0.01 M sodium hydroxide were used. Pellets were prepared by compacting 100 mg of sample at a pressure of 5 tonnes using a suitable hydraulic press. The experiment was performed at 37°C.

Suitable conditions were chosen which would lead to approximately 10–20% release of the drug substance over a reasonable time period. For the final analysis, samples were taken at 10-, 20-, 30-, 40-, 50-, and 60-minute intervals with a total release of ca. 15% of the drug. Samples were measured by UV-visible spectrophotometry at a wavelength of 240 nm using a Specord 205 spectrophotometer (Analytik Jena, Jena, Germany). The intrinsic dissolution rate (mg/ml/cm^2^) was calculated as the slope of the best-fitting line (mg/min) divided by the surface area of the drug substance (cm^2^).

*In vitro* diffusion experiments were performed using a suitable Franz cell apparatus (Hanson Research, Chatsworth, USA) comprising 6 jacketed, diffusion cells connected by suitable tubing to a heated reservoir of fluid which was kept at a constant temperature of 32°C using a ScanVac SHC 2000 temperature bath (Labogene, Lynge, Denmark). Each diffusion cell contained a magnetic stirring bar and approximately 7 ml of a collector medium comprising a mixture of 50 : 50 methanol/water (v/v). Porafil® membranes (Macherey-Nagel, Düren, Germany) made from regenerated cellulose and having a porosity and diameter of 0.45 *μ*m and 25 mm, respectively, were employed in all tests. 300 mg of the cream sample were used for each diffusion cell. Sampling was performed at intervals of 60, 120, 180, 240, and 300 minutes and the samples were measured using a validated HPLC procedure. The HPLC method is comprised of a Spherisorb ODS 2, 150 × 4.6 mm column packed with 5 *μ*m particles and a mobile phase comprising methanol, 10 g/L phosphoric acid, purified water, and acetonitrile at a ratio of 10 : 20 : 20 : 50 v/v/v/v. The flow-rate, column-oven temperature, and detector wavelength were 2.0 ml/Min, 25°C, and 235 nm, respectively. The method was run isocratically for 15 minutes and an injection volume of 15 *μ*l was used.

## 3. Results and Discussion

### 3.1. FTIR and XRPD Analysis

**Figure 2:**
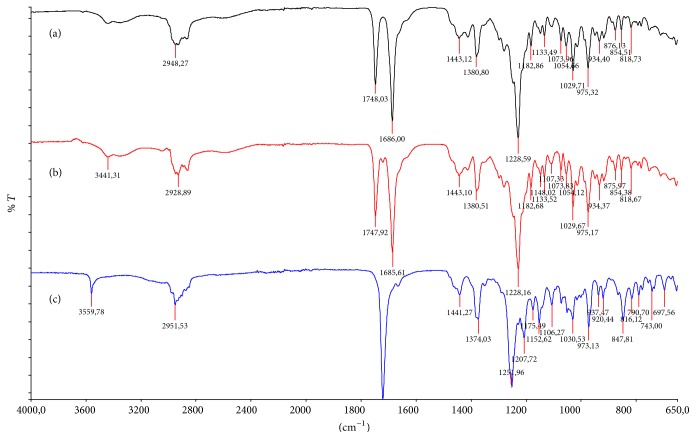
FTIR spectra of FA from Manufacturers A, B, and C demonstrating major differences in the region 1600 cm^−1^ to 1800 cm^−1^. Spectra (a) and (b) correspond to polymorphic Form III and show major differences as compared to spectrum (c) which shows polymorphic Form I.

The characterisation of FA has been described in a number of patents as well as in the mainstream scientific literature. FTIR spectra and XRP diffractograms of each of the polymorphic forms of FA have previously been described [17–19]. The most significant differences between the FTIR spectra of these forms are observed in the region 1650 cm^−1^ to 1750 cm^−1^. Form I shows a single stretching band at ≈1720 cm^−1^, whilst Form II shows a band at ≈1720 cm^−1^ and an additional stretch at ≈1697 cm^−1^ which is not present in the other forms. Form III shows two very distinct bands at approximately 1748 cm^−1^ and 1688 cm^−1^ which are also not present in the other forms. A comparison of the literature data with the FTIR data of FA from the commercial samples shows that FA from Manufacturer A is of Form III, FA from Manufacturer C is of Form I, and FA from Manufacturer B is comprised predominantly of Form III but is not of pure crystalline form. It contains significant quantities of either Form I or Form II, which can be seen by the presence of an extra absorption band at ≈1720 cm^−1^ (Figure 2).

**Figure 3:**
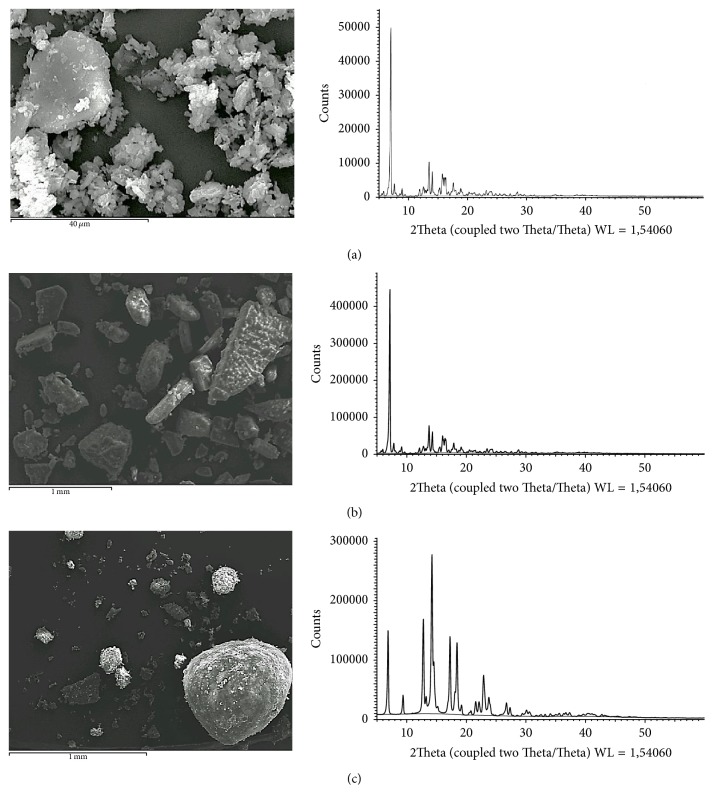
Scanning electron micrographs and X-ray powder diffractograms of FA from Manufacturers A (micronised) and B and C (both nonmicronised). Manufacturers A and B show identical reflexes corresponding to polymorphic Form III. The diffractogram of FA from Manufacturer C shows major differences to A and B and corresponds to polymorphic Form I as described in the literature.

Powder X-ray diffraction analysis of FA from Manufacturers A, B, and C (Figure 3) demonstrates that A and B are of similar crystal form (Form III), since both diffractograms are comparable for all significant reflexes. In contrast, the diffractogram of FA from Manufacturer C shows significant differences to A and B and is comparable with the literature data for Form I. The XRPD data agrees with the results of the FTIR analysis. Scanning electron micrographs of FA from each of the manufacturers show differences in morphology (Figure 3).

### 3.2. Stability Studies

#### 3.2.1. Stability Studies

**Figure 4:**
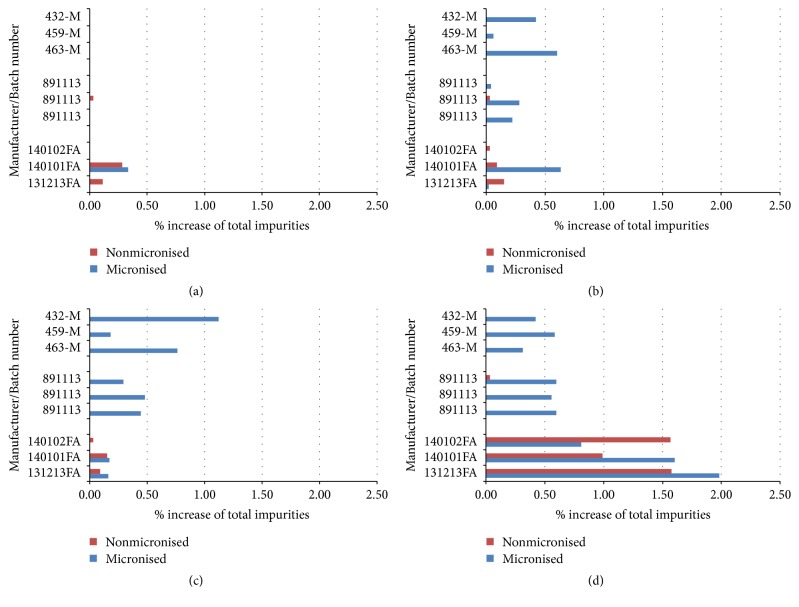
Results of stability studies of FA performed at 5°C ± 3°C (a), 25°C/60% RH (b), and 40°C/75% RH (c) as well as under photolytic stress conditions as described in ICH Q1B (d).

The study results (Figures 4(a), 4(b), and 4(c)) indicate that the stability of FA is temperature dependent. The greatest stability is observed at 5 ± 3°C, with the stability decreasing with increasing temperature. No significant difference in stability was observed between polymorphic Form I (Manufacturer C) and Form III (Manufacturers A and B) under ICH conditions. The data indicated that micronisation of the API has a negative effect on the stability of FA, as demonstrated by the higher levels of impurities observed in this material as compared to the nonmicronised API. This is likely due to the reduction of the particle size and corresponding significant increase of the specific surface area of the substance after micronisation. XRPD and FTIR analysis confirmed that the polymorphic form of FA does not change during micronisation or storage.

#### 3.2.2. Stress Tests

The ICH stability studies described above demonstrate that there are no significant differences in stability between polymorphic Forms I and III under the chosen conditions. However, under harsher conditions both light and humidity give rise to degradation in FA. The photostability of FA was examined according to ICH guideline Q1B, whereby the samples were illuminated for 1.2 million lux hours with energy of 200 watt hours/m^2^. The humidity test was performed by placing fusidic acid in an open container and storing at 60% relative humidity for 6 months. The results of both tests indicated that FA from Manufacturer B was significantly less stable under these conditions than FA from either of the 2 other manufacturers (Figure 4(d)). These results are however unlikely to be relevant for the stability of the drug substance in a medicinal product since the product would never be exposed to such harsh environmental conditions during manufacture, transport, or storage.

### 3.3. Intrinsic Dissolution Rate

In order to compare the solubility properties of FA from each of the 3 manufacturers, the intrinsic dissolution rates of each sample were analysed according to Ph. Eur. 2.9.29 using the rotating cylinder apparatus. Preliminary experiments were performed in order to investigate the effect of rotation speed (50, 100, and 150 rpm) and press tonnage (3, 5, and 7 tonnes) on the release rate of the substance. The rotation speed was found to have a significant effect, with an increased rotation speed leading to a more rapid release rate. The press tonnage had no effect on the release rate. FTIR spectra of the samples taken before and after pressing showed that no phase change occurred during the preparation of the pellets.

The measured release rates were 0.42, 0.36, and 0.30 mg/min/cm^2^ for Manufacturers A, B, and C, respectively. Statistical analysis of the data was performed using a nonparametric confidence interval procedure related to the Mann–Whitney rank test [26]. The analysis showed no significant difference between the IDR profiles of FA, indicating that the samples have comparable intrinsic dissolution rates. This is in good agreement with previous literature results which found similar intrinsic dissolution rates for Forms I, II, and III [19].

### 3.4. *In Vitro* Release Profile from Fusicutan plus Betamethasone Cream

The results of the intrinsic dissolution tests indicated that the investigated samples show comparable dissolution rates in the model system used for this test. This data provides useful clues about the comparability of the solubility properties of the measured samples. It can however not easily be extrapolated to the release rate of the drug substance from a semisolid drug product. In semisolid systems, the drug substance is very often suspended in a complex matrix, with only a fraction of the total quantity of drug substance being dissolved in solution. In order to compare the release rates of drug substances from semisolid preparations, an* in vitro* penetration test is often employed. One of the most commonly employed versions of such a test uses a vertical diffusion cell, or Franz cell, comprising a jacketed, vertical diffusion cell connected by appropriate tubing to a heated reservoir of fluid which is continuously pumped around the system to ensure a constant temperature of 32°C, thus mimicking the temperature at the external skin surface. This model was employed in the present case. Quantitative analysis was performed using a validated HPLC procedure with UV detection.

**Figure 5:**
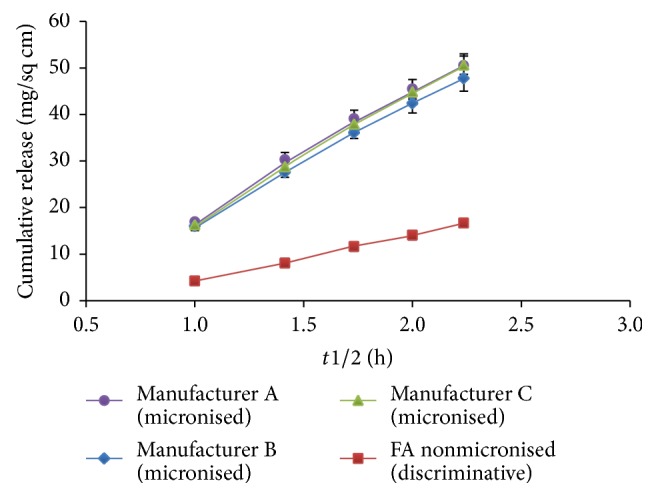
*In vitro* release profiles of FA from Fusicutan plus Betamethasone cream showing statistically comparable release rates for FA from all 3 drug substance manufacturers.

In order to demonstrate the discriminatory power of the procedure, that is, the ability of the procedure to detect differences in the release rates of API from different cream formulations, a batch of cream was prepared which contained nonmicronised FA (note: FA is normally present in the finished formulation in its micronised form). The release rate for this batch was significantly slower than for the test batches, which all contained micronised material. The slower release rate can be attributed to the larger particle size of the FA crystals which take longer to dissolve. This indicates that the analytical procedure is capable of discriminating between batches with different release rates.  Figure 5 shows the results of the* in vitro* diffusion test. A statistical analysis of the release rates of FA from each of the test batches of cream was performed using the same procedure as described in Section 3.3 [26]. The release rates were not significantly different according to this test.

## 4. Conclusions

The results of the present study indicate that there are at least 2 polymorphic forms of fusidic acid (I and III) currently available on the commercial market. Under refrigerated, real-time, and accelerated stability conditions, the API from all 3 sources employed in this study were found to have comparable stabilities when packaged under an atmosphere of nitrogen in an air-tight, light-protective container. The stability of FA was found to be temperature dependent with the greatest stability being observed at 5°C ± 3°C. Micronisation was found to reduce the stability of FA as compared to nonmicronised material. Under stress conditions, the API from Manufacturer B was found to be significantly less stable than material from either of the other 2 sources. This instability is unlikely to be related directly to the stability of the predominant polymorphic form of the material (Form III), since Manufacturer A is of the same form and is stable. The instability may be related to the fact that the material is not of pure polymorphic form. The current study demonstrated that material from Manufacturer B contains substantial quantities of another polymorph which may influence the overall stability of the material. This issue was however not within the scope of the current study and requires further investigation.

The intrinsic dissolution rates of each of the drug samples, as well as their* in vitro* diffusion rates from the marketed topical product Fusicutan plus Betamethasone, were analysed and found to be statistically comparable. The data indicates that polymorphic Forms I and III found in FA samples from the investigated manufacturers have similar solubility properties and can be used interchangeably in semisolid drug product formulations without affecting the safety and efficacy of those formulations.
